# A measurement method for mental health based on dynamic multimodal feature recognition

**DOI:** 10.3389/fpubh.2022.990235

**Published:** 2022-12-23

**Authors:** Haibo Xu, Xiang Wu, Xin Liu

**Affiliations:** ^1^Center for Mental Health Education and Research, Xuzhou Medical University, Xuzhou, China; ^2^School of Management, Xuzhou Medical University, Xuzhou, China; ^3^Institute of Medical Information Security, Xuzhou Medical University, Xuzhou, China; ^4^School of Medical Information and Engineering, Xuzhou Medical University, Xuzhou, China

**Keywords:** mental health assessment, emotion recognition, interactive assessment scale, video feature extraction, deep learning

## Abstract

**Introduction:**

The number of college students with mental problems has increased significantly, particularly during COVID-19. However, the clinical features of early-stage psychological problems are subclinical, so the optimal intervention treatment period can easily be missed. Artificial intelligence technology can efficiently assist in assessing mental health problems by mining the deep correlation of multi-dimensional data of patients, providing ideas for solving the screening of normal psychological problems in large-scale college students. Therefore, we propose a mental health assessment method that integrates traditional scales and multimodal intelligent recognition technology to support the large-scale and normalized screening of mental health problems in colleges and universities.

**Methods:**

Firstly, utilize the psychological assessment scales based on human-computer interaction to conduct health questionnaires based on traditional methods. Secondly, integrate machine learning technology to identify the state of college students and assess the severity of psychological problems. Finally, the experiments showed that the proposed multimodal intelligent recognition method has high accuracy and can better proofread normal scale results. This study recruited 1,500 students for this mental health assessment.

**Results:**

The results showed that the incidence of moderate or higher stress, anxiety, and depression was 36.3, 48.1, and 23.0%, which is consistent with the results of our multiple targeted tests.

**Conclusion:**

Therefore, the interactive multimodality emotion recognition method proposed provides an effective way for large-scale mental health screening, monitoring, and intervening in college students' mental health problems.

## 1. Introduction

Most symptoms of early mental health diseases are subclinical, and it is hard to give a definitive diagnosis (1–3). Affected by COVID-19 in recent years, the mental health problems of college students have become more serious. As related research reports that 54% of students had moderate or severe psychological problems, 29% had moderate-to-severe anxiety, and 17% had moderate-to-severe depressive symptoms. So it is an urgent problem how to make a clear and accurate evaluation of degrees of mental health illnesses of college students and give corresponding or personalized treatment plans in the state of COVID-19 (4, 5).

The traditional mental health evaluation method is the psychological scale, such as Symptom Checklist 90 (SCL-90) (6), Patient Health Questionnaire-9 (PHQ-9) (7), 16-item Quick Inventory of Depressive Symptomatology and Self-Report (QIDS-SR16) (8), Depression Anxiety and Stress Scale (DASS-21) and so on (9). DASS-21 is one of the most widely used and preferred scale versions (10). Although the convergent validity of DASS-21 is crucial, there is still a lack of testing for its convergent validity (11–13). Compared with children and adults, college students have colonial existence frequently, which means that mental health problems are inevitably caused by contradictions between people (14–16). Moreover, some college students failed to provide accurate and timely responses to inquiries about mental health assessments (17–19), which leads to the results of psychological assessments being inaccurate (20, 21). Using objective, intelligent and efficient methods except psychological scales to assess the college student's mental health degree is an urgent problem.

With the rapid development of information technology, Artificial Intelligence (AI) can mine the deep correlation of multiple layers of psychological data, which has attracted widespread attention (22–24). Related studies have shown that individuals with mental health problems have abnormal physical characteristics (mood, voice, body shape, etc.) (25, 26). For example, Simcock et al. examined the association between psychological problems in early adolescence and facial emotion recognition, showing that anxiety and depression were significantly associated with angry expressions (27). Arevian et al. (28) evaluate the feasibility of collecting speech samples from people with serious mental illness and explores the potential utility of tracking changes in clinical state over time. Santore (29) explores the relationship between repetitive behaviors and obsessive-compulsive disorder and autism by studying adolescents with autism spectrum disorders. Therefore, combining multi-modal data and intelligent information technology can effectively assist in evaluating individuals' mental health problems, which is more effective for large-scale college students' mental health screening.

To provide a more objective and effective evaluation method for college students' normalized mental health, we propose a method for assessing psychological problems that combine multimodal data with traditional scales, intelligently correcting the evaluation results by capturing the unconscious characteristic data of individuals and giving the evaluation results quickly and accurately. The main contributions are as follows: propose the psychological assessment scales survey of college students utilizing a human-computer interaction measure model; collect multiple characteristic data for multimodal emotion recognition to assist mental health representation; the effectiveness of the proposed method is evaluated through multiple dimensions, and a comparative experimental analysis is given. The results show that the proposed mental health assessment method performs better; a complete mental health assessment system for college students has been constructed, providing an essential method for normalized large-scale psychological problem screening.

## 2. Materials and methods

### 2.1. Study procedures and participants

The Ethics Committee approved all procedures of Xuzhou Medical University, and all materials are available upon request. Furthermore, all participants were informed about this study's purpose and what was being collected, informed of their rights, and provided informed consent before data collection. The study participants were all college students at Xuzhou Medical University. The specific process is as follows: The Mental Health Center of Xuzhou Medical University publishes the recruitment information through the intranet of Xuzhou Medical University, and students who voluntarily participate in this study sign the written informed consent and enter the test through the evaluation link: https://www.bowenzz.top:8080/. The system supports participants' use of mobile phones or computers for testing. The evaluation content includes online questionnaires, facial emotion feature collection, and audio feature collection. The detailed data collection and modeling process are in Section Measurement instruments. Participants need to follow the prompts to complete the corresponding question and answer. The interactive questionnaire system can quickly give the measured results according to the collected multimodal information.

The experimental sample comprised 1,500 college students with the random sampling method, including undergraduates and students aged 17–34. The mean age was 19.20 years, and the standard deviation was 2.31. The proportion of female participants is 60%, and the proportion of male participants is 40%. Some students may have varying severity of mental health problems affected by managing the epidemic blockade for a long time. This study has been supported by the Key Science and Technology Program of Xuzhou: Research on key technologies of psychosocial services for normalized epidemic prevention and control.

### 2.2. Measurement instruments

#### 2.2.1. Design of data acquisition system

The questionnaire used the DASS-21 is a 21-item self-report questionnaire that assesses recent experiences of stress (e.g., “I found it hard to wind down”), anxiety (e.g., “I felt close to panic”), and depression (e.g., “I felt that I had nothing to look forward to”). Each 7-item subscale is rated on a 4-point Likert scale ranging from 0 (Did not apply to me at all) to 3 (Applied to me very much). Higher scores represent more significant symptomology.

An interactive questionnaire system has been designed to collect experimental sample data. While conducting human-computer interaction questionnaires, the system can collect interactive videos of participants, extract corresponding facial and audio signals and perform multimodal emotion recognition. In addition, considering personal privacy, the system anonymizes and encrypts the personal information and the collected videos.

In the modeling stage of the multimodal emotion recognition model, we mainly use neural network architectures to train corresponding prediction models, applying transfer learning and ensemble learning methods to handle different types of data, including video signals, emotion detection for audio signals, and emotion recognition for text information. Finally, the highly reliable multimodal emotion recognition detection model was constructed and completed.

#### 2.2.2. Interactive scales

**Figure 1:**
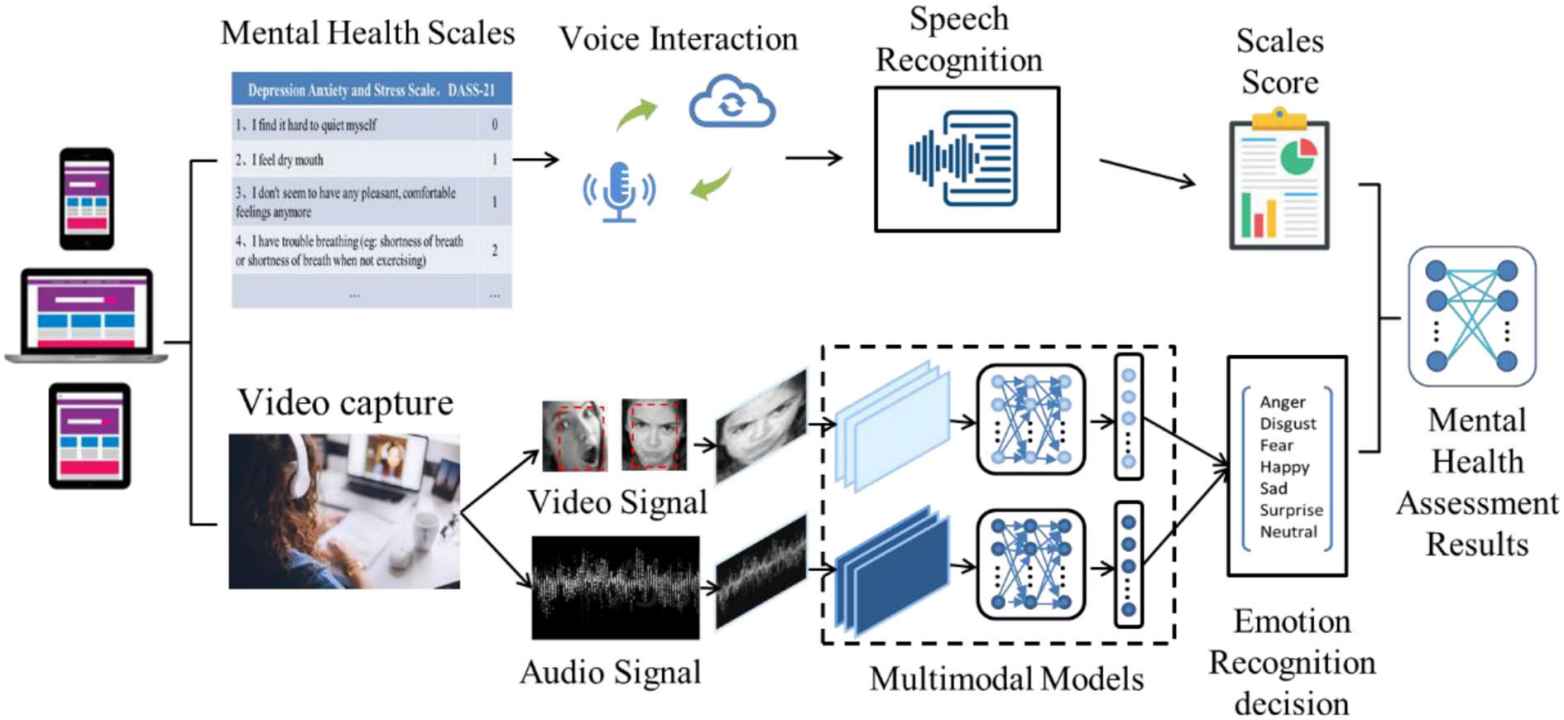
Interactive multimodal emotion recognition scales system. The system includes two parts: an interactive questionnaire process and a multimodal emotion recognition process.

As shown in Figure 1, the Interactive Assessment Scales (IAS) is a video and voice interactive question answering system framework that provides composite prompt function, including verbal and non-verbal cues. IAS adopts a modular architecture design consisting of three main modules: a voice interaction module, a video acquisition module, and a statistical analysis module. In the voice interaction module, the synthetic assessment scales are designed to realize the answering function in interactive scales using text-to-speech TTS technology. The video acquisition module integrates face detection, facial feature extraction, and audio emotion analysis functions. In the statistical analysis module, we design non-voice prompting functions, including natural language recognition, text emotion detection, and corresponding functions of scale information statistics. The evaluation process is as follows: First, the system uses voice interaction to describe the multiple-choice questions of the assessment questionnaire to the user. The user answers by saying one of the possible results, and each question can only be answered by choosing from a fixed set of answers. Then, the system checks whether it matches the alternative answer through natural speech recognition and will prompt the user to repeat the answer if the answer cannot be recognized. Otherwise, the answer is recognized, and the user is allowed to answer the following question after confirmation. In this process, by collecting the video and audio signals of the user's answer, we can analyze the possibility of true and false answers according to the emotional state reflected by the user to adjust the confidence of the evaluation score to match the user's actual psychological state as much as possible.

#### 2.2.3. Multimodal emotion models

Since single-modal information influenced by various noises can not fully reflect emotional states, we proposed a multimodal hybrid deep model, which utilizes ensemble learning to train facial video and audio data of emotion recognition models. The facial video model is based on the Xception method (30), a deep convolutional neural network architecture involving depth-wise separable convolution. The audio emotion detection model is a temporally distributed convolutional neural network; its main idea is to apply a rolling window along the log-mel-spectrogram. These windows are the entry points of a convolutional neural network, and the output of each convolutional network will be fed to a 2-unit LSTM (Long Short Term Memory) to learn long-term contextual dependencies (31). The multimodal data fuses the potentially shared information of each modal and compares the emotion recognition results using an enumeration weighted decision fusion strategy based on statistical rules and probability theory.

The loss function can measure the contributions of video and audio modalities, and we choose to derive a loss function that updates parameters according to the contributions of different modalities during back propagation. The derived loss function integrated with a deep neural network is defined in Equation (1), where *l* is a measure of the difference between the prediction and the ground truth, *N* is the number of modalities, *x*_*i*_ is the output feature of the *n-*th modality, and ω_*i*_ is the fully connected network regarding the weight of the nth modality, while *y* is the ground truth.


(1) $$\begin{array}{l} Loss=\Sigma_{i=1}^{N}l\left(x_{i},w_{i},y\right) \end{array}$$

In the classification task of multimodal emotion recognition, video and audio embedding patterns are helpful to predict real emotions. The cross-entropy loss defined in Equation (2) is chosen as the standard function.


(2) $$\begin{array}{l} l\left(x\right)=-\frac{1}{n}\Sigma_{x}\left[y\;logx+\left(1-x\right)\left(1-y\right)\right] \end{array}$$

A multimodal emotion recognition model was used to identify participants' emotions during the assessment process. The participants were summarized in a time series according to the identified expression categories: neutral, happy, angry, surprised, sad, disgust, and fear. The questionnaire covers three mental health characteristics: stress, anxiety, and depression. These characteristics correlated with the multimodal emotions that participants exhibited during the assessment. There was also a correlation with negative emotions that participants displayed during the assessment. And the correlation between the degree of modeling analysis, as close as possible to their actual mental health status.

## 3. Results

### 3.1. Experimental data collection and processing

The investigation began after more than a month of quarantine and lockdown at the university, and the relevant authorities approved the approval of the experiment. Participants were informed about the purpose of the experiment and what was being collected, informed of their rights, and provided informed consent before data collection. Participants provided demographic information (e.g., age and gender) and emotional state information, and data was collected and analyzed in a secure environment and under strict confidentiality. The mean age of the sample was 19.2 years, and the standard deviation was 2.31. After dis-aggregation, the proportions for each age group were 77.14% before age 20 and 22.86% over 20 years old. Among them, female participants accounted for 60%. Participants then completed an interactive DASS-21 questionnaire and corresponding video signal acquisition. In a word, this study used more complete data types, including structured and unstructured data, to ensure the accuracy and reliability of the analysis results. The DASS-21 questionnaire analyzes the data, and then the multi-modal information is input for emotional model recognition. Finally, the results of the two algorithms are combined to obtain the final evaluation result.

After students completed the online questionnaire, we performed missing value screening and deletion on the collected data. And calculate statistics based on criteria for demographic variables. Cronbach's alphas were calculated as an indicator of internal consistency. We calculated means, standard deviations, skewness, kurtosis, and corrected item-total correlations for individual items. A three-factor model was used to test structure validity and measurement invariance of the DASS-21. Furthermore, the three one-factor models for each subscale were tested.

In the emotion recognition process, video and audio data embedding contribute to the final prediction result, and the loss function can measure the contributions of these two modalities separately. We choose to derive a loss function that updates parameters based on the contributions of different modalities during backpropagation. In classifying patients with emotion recognition, cross-entropy with logits is used, which is a commonly used method in multi-classification tasks. The cross-entropy loss is chosen as the standard function in the mental health assessment task. The multi-trait-multi-method matrix involved six traits: neutral, happy, angry, surprise, sad, disgust, and fear; two methods were involved, including the DASS-21 subscale and the multimodal emotion recognition model. Calculate the Pearson correlation coefficient between the predicted value and the DASS-21 score to obtain the correlation between different methods measuring the same trait and the correlation between different traits measured by the same method.

We pre-train the multimodal emotion recognition model with the Ryerson Audio-Visual Database of Emotional Speech and Song (RAVDESS) and the FER2013 Kaggle Challenge dataset (32, 33). The RAVDESS database contains 7,356 files of pure audio, audio-video and pure video, of which 1,440 are audio files. The audio included calm, happy, sad, anger, fear, surprise, and disgust, produced at two levels of emotional intensity (regular and intense), with an additional neutral tone. The FER2013 Kaggle Challenge dataset consists of 48 × 48 pixel grayscale images of human faces and contains 28,709 examples. Including seven emotions: anger, disgust, fear, happiness, sadness, surprise, and neutral.

### 3.2. Exploratory factor analysis

The results of the interactive mental health assessment are shown in Table 1. In the depression subscale, the proportion of college students who showed no symptoms, mild symptoms, moderate symptoms, severe symptoms, and extreme symptoms was 67.07, 9.96, 10.56, 2.86, and 9.56%. In the anxiety subscale, the proportions were 34.86, 17.00, 11.35, 8.90, and 27.89%. In the stress subscale, the proportions were 54.91, 8.76, 16.33, 13.55, and 6.44%.

**Table 1:** Interactive DASS-21 descriptive statistics.

	**Total**	**Age**	**Normal**	**Mild**	**Moderate**	**Server**	**Extreme**	**Confidence**
**DASS-21**	1,500	19.20(2.31)						0.927
Male	600	18.99(2.10)						0.935
Female	900	19.35(2.42)						0.919
**DASS-21-S**			54.91%	8.76%	16.33%	13.55%	6.44%	0.840
Male	600	18.99(2.10)	55.56%	7.63%	15.42%	14.26%	7.13%	0.858
Female	900	19.35(2.42)	54.39%	9.57%	16.91%	13.13%	6.01%	0.825
**DASS-21-A**			34.86%	17.00%	11.35%	8.90%	27.89%	0.803
Male	600	18.99(2.10)	33.50%	16.42%	12.60%	8.96%	28.52%	0.809
Female	900	19.35(2.42)	35.71%	17.35%	10.57%	8.90%	27.47%	0.799
**DASS-21-D**			67.07%	9.96%	10.56%	2.86%	9.56%	0.835
Male	600	18.99(2.10)	62.69%	10.12%	12.11%	3.98%	11.11%	0.848
Female	900	19.35(2.42)	69.97%	9.90%	9.45%	2.11%	8.57%	0.820

DASS-21-S, means the stress of DASS-21; DASS-21-A, means the anxiety of DASS-21; DASS-21-D, means the depression of DASS-21.

The Cronbach alpha test results in a DASS-21 value of 0.927, a DASS-21-S value of 0.840, a DASS-21-A value of 0.803, and a DASS-21-D value of 0.835. Cronbach alpha values were >0.7 in all cases, indicating that all collected data achieved internal consistency within a large group and subgroups. Because of the non-normal distribution of anxiety, depression, and stress scores in this sample, the median was taken as the most reliable indicator of the midpoint of the sample. The median scores for stress, anxiety, and depression were 6, 4, and 2, respectively. After categorizing outcomes by the midpoint, 45.11% of participants were higher than the median stress score, 48.17% were higher than the median anxiety, and 46.18% were higher than the median depression variable. When divided into three score scales, the mean and standard deviation of stress, anxiety, and depression factors are as follows: 7.29 (SD = 6.44), 6.52 (SD = 5.75), and 4.54 (SD = 5.47), respectively.

### 3.3. Multimodal emotion recognition

**Figure 2:**
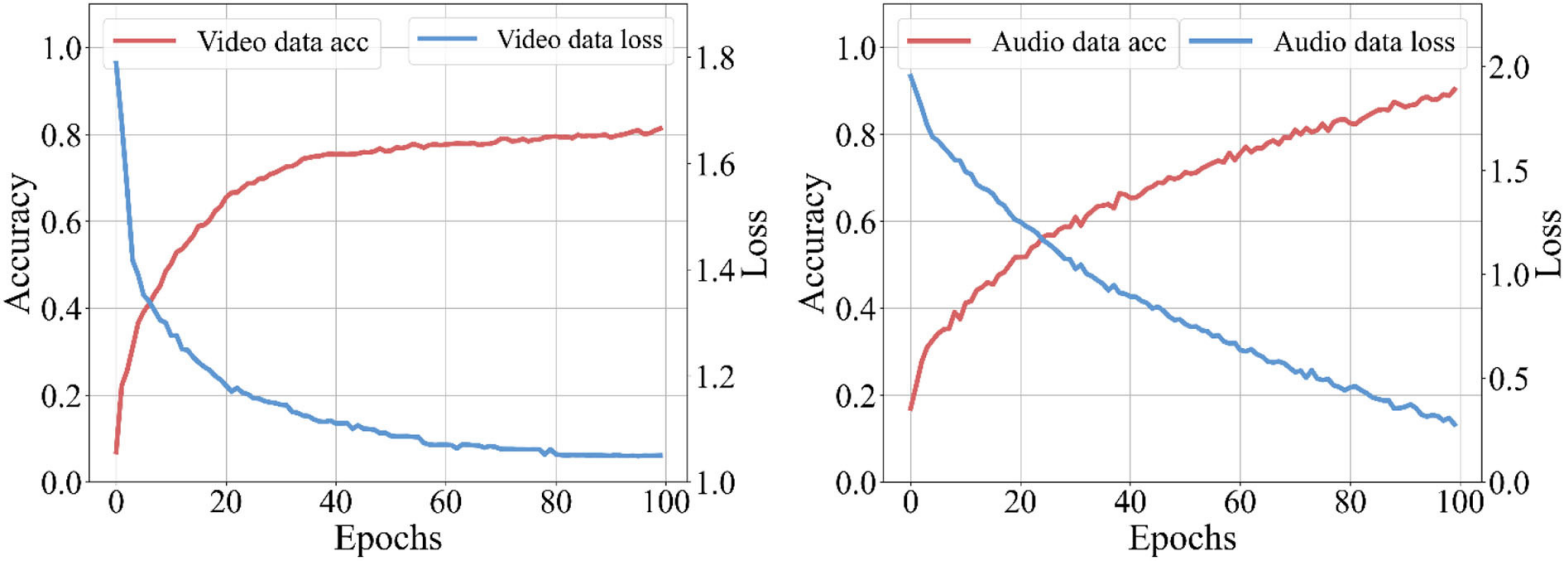
Recognition accuracy and loss of the emotion recognition model.

To verify the performance of the multimodal emotion recognition model, we compare the proposed model with other innovative models in video and audio emotion recognition. As shown in Figure 2, the figure shows the recognition accuracy and loss of the multimodal emotion recognition model. The model's accuracy in video and audio reaches 82.3 and 90.2%, which can fully meet the recognition requirements of real scenes.

Table 2 shows the performance of multimodal emotion recognition on RAVDESS compared to Attention-BLSTM-FCN (31), HSF-CRNN (34), and CNN TF Att.pooling (35). Furthermore, compared to KeyFrame (36), ESR-9 (37), and PREP (38) on the FER2013 dataset. In audio emotion recognition, our model outperforms the state-of-the-art models; in video emotion recognition, our model is slightly worse than ESR-9 and outperforms other models. Overall, our model has outstanding performance in video and audio emotion recognition.

**Table 2:** Performance comparison with other models.

**Features**	**Models**	**Accuracy (%)**
**Audio**	Attention-BLSTM-FCN	72.5
	HSF-CRNN	81.3
	CNN TF Att.pooling	80.5
	Our model	90.2
**Video**	KeyFrame	79.1
	ESR-9	85.1
	PREP	75.7
	Our model	82.3

**Figure 3:**
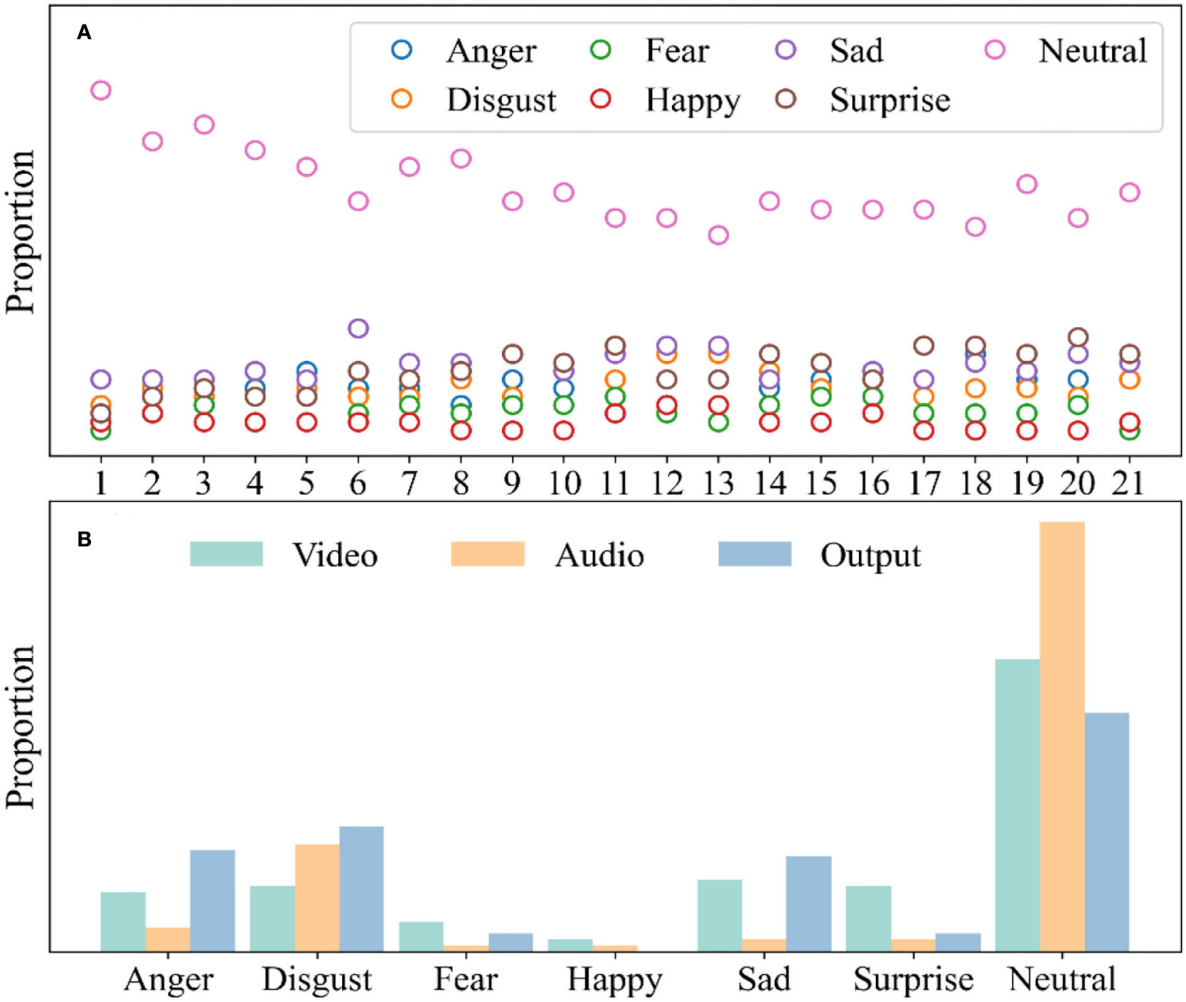
Evaluation of multimodal emotional classification. **(A)** Time series analysis in video emotion recognition; **(B)** The participants' emotion distribution.

Analyzing the participants' video and emotional audio signals during the answering process, it conducted a summary and preliminary analysis, as shown in Figure 3. Figure 3A shows the time series statistics of the participants' emotional changes in the questionnaire process. It can be seen from the figure that in the process of answering, most people showed a stable emotional state, while others showed relatively unstable. This proportion is relatively close to that of students with severe or above various types.

As shown in Figure 3B, the multimodal emotion recognition model identified all emotion distributions in the participant assessment process. Studies have shown that people with poor mental health are more likely to produce and express negative emotions under neutral stimuli (39). Compared with the previous scale questionnaire, most participants' emotions were captured as neutral, and only a few were captured as negative. However, if all negative emotions are counted, the proportion is relatively large. Therefore, it can be considered that there is a real connection between the emotions identified by multimodality and mental health status.

### 3.4. Correlation analysis

**Figure 4:**
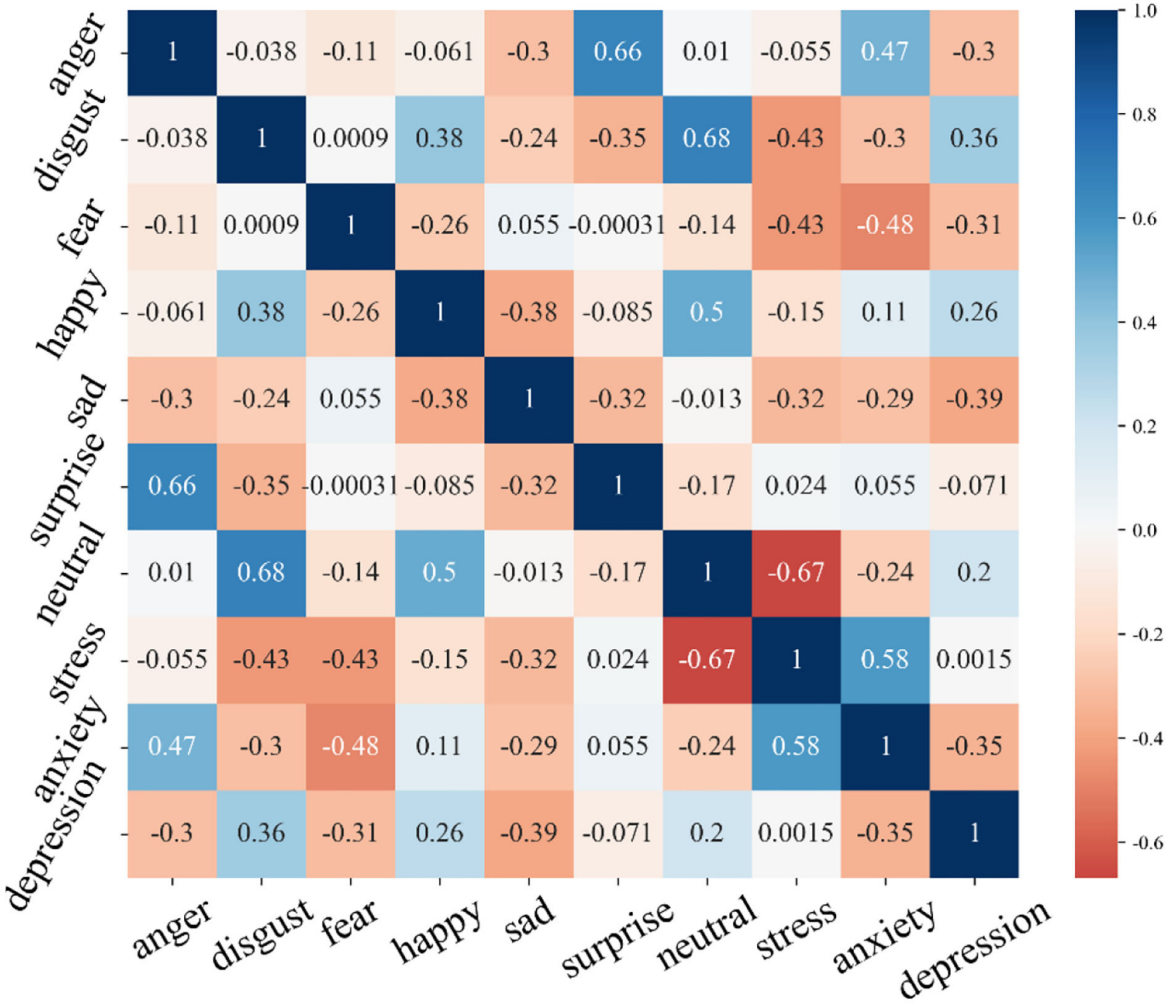
The multitrait-multimethod matrix involved a zero-order correlation matrix between six trait variables.

The Pearson correlation coefficient between the predicted value and the corresponding subscale's actual score was calculated, and the standard validity analysis was carried out. We use a multi-feature-multi-method matrix to explore the structural validity of the multimodal sentiment prediction model. The matrix involves three traits feature attributes and seven emotion attributes of 2 methods, including the DASS-21 subscale and the emotion recognition model. The Pearson correlation coefficient between the predicted value of emotion recognition and the DASS-21 score was calculated. Figure 4 presents the zero-order correlation matrix between the variables. It is divided symmetrically on the diagonal line. Numbers indicate the correlation between emotional traits and different psychological symptoms, ranging between [−1, 1]. A positive and larger value indicates a positive correlation between attributes, and a negative and smaller value indicates a negative correlation between the attributes. At the same time, the color features in the graph are also used to represent the correlation features between the attributes. The results show that there is some correlation between the degree of mental health symptoms and multimodal emotions, especially some emotion with negative attributes. These results demonstrate that the proposed method can effectively establish the association with mental health status. However, the correlation degree of related attributes is not particularly significant, so there is still a deep correlation between various psychological symptoms and emotional features to be explored.

**Figure 5:**
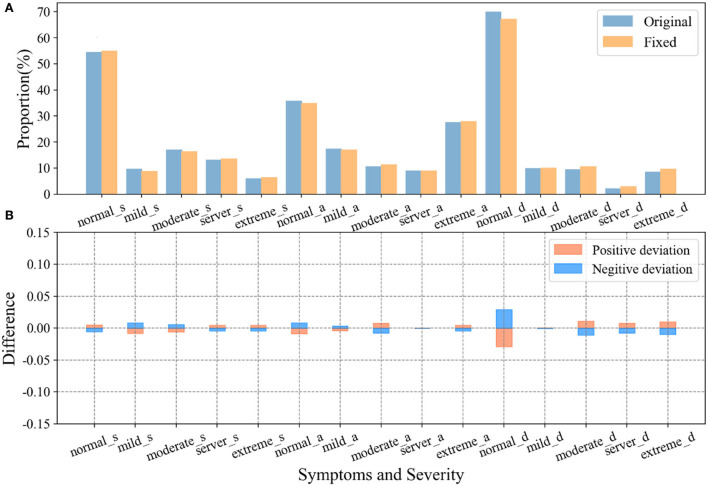
Evaluation results after correction and comparison with those before correction. **(A)** DASS-21 scales the proportion of different severity of stress, anxiety, and depressive symptoms before and after correction; **(B)** Bias in the proportion of symptom severity before and after correction.

Based on the above analysis, the mental health of participants who exhibited negative emotions in the evaluation was corrected by establishing the relationship between the questionnaire and multimodal emotions. Figure 5 shows the evaluation results after correction based on emotion recognition and the comparison with before correction. Figure 5A shows the proportion of symptoms in the three subscales of DASS-21, including the proportion of the original scores and the proportion of the revised scores. Figure 5B shows the deviation of the results of the two proportions of three symptom severity before and after correction. It can be seen that the emotion recognition correction has the most significant impact on the results of the extreme of each symptom, which is related to the fact that the extreme participants are more likely to show obvious emotional characteristics.

## 4. Discussions

The mental health problems in college students have traditionally been detected based on scores on the mental health questionnaire alone. However, researchers may be unable to accurately provide mental health counseling and treatment due to withholding facts and psychologists making misjudgments. Furthermore, previous studies have shown that the DASS-21 scales are biased in measuring the mental health of college students (40, 41). Therefore, this study combines the interactive mental health questionnaire with an intelligent algorithm-based multimodal emotion recognition model. To optimize the scale results by multimodal emotion recognition of video signals, we propose a very suitable evaluation system that adopts a multi-factor structure to measure the mental health of college students, effectively assessing the mental health degree of college students.

A reported cross-sectional survey sheds light on stress, anxiety, and depression among college students in the COVID-19 pandemic (42). Results of the study showed that anxiety factors had the lowest prevalence among students (19.7%) in long-term closed environments, while the highest prevalence of anxiety among general college students was previously reported (47.3%). In the context of the COVID-19 epidemic, college students' direct psychological responses indicated that students had lower rates of stress but higher rates of anxiety and depression. This study's results also indicate significant differences in anxiety rates among students using the interactive multimodal emotion recognition DASS-21 scale. The student's anxiety rates increased significantly from 47.3% using the DASS-21 to 48.1% using this method, while stress and depression up to 36.3 and 23.0%, respectively. Many students may miss diagnoses because of their mental health levels when using the DASS-21. This means that the validity of the DASS-21 scale needs to be explicitly validated before it is applied to each specific setting.

This study further clarified the association between mental health and emotions. Consistent with previous research on emotion-evoking conditions, facial emotion and voice emotion can also be used to differentiate between mentally ill and healthy individuals under neutral conditions, with those with poorer mental health more likely to produce than healthy individuals and expressing negative emotions under neutral stimuli (43). Although each model had significant standard validity, it is worth noting that the standard validity of the depression and anxiety models was lower than that of the other symptom dimensions. This is because individuals with depression and anxiety disorders may have different subtypes, leading to various emotional manifestations, resulting in slightly lower legal validity. Related studies have also pointed to differences in performance between individuals with multiple symptoms and those with only depression or anxiety disorders (44, 45). Therefore, our study shows that different psychological symptoms of mental health may have different emotional information, which can correspond to the interactive DASS assessment process. These scores are detailed and refined. Future research needs to explore each symptom's unique expressions of dimensions and underlying emotional mechanisms.

The study also has some limitations. First, the number of samples is insufficient to meet the needs, and some calculations based on statistical theory may be biased. Although age and gender were balanced, the participants' shared experiences may also have contributed to some sampling bias. Second, the accuracy and applicability of the multimodal emotion recognition model need to be improved. This is mainly the choice of a machine learning algorithm to ensure that it can match the corresponding data set to ensure that the applicability will improve the results. Third, considering the purpose of the study, we used the DASS-21, whose subscales had very high correlations, which resulted in low discriminant validity. Further work should consider comorbidities between symptoms and strive to obtain corresponding unique emotions for each symptom. Furthermore, another potential use of this comprehensive model could be in predicting the risk and timing of mental health events in graduate students. Finally, by tracking the progress of students' emotions over time, understanding the mental health changes in different periods is one of our future research directions.

## 5. Conclusions

This study explores a multimodal emotion recognition model using an interactive assessment method based on the DASS-21 questionnaire for detecting the prevalence of anxiety and depressive symptoms in college students during the COVID-19 pandemic. The experimental results show that the interactive multimodal emotion recognition method has high reliability and satisfactory results. This study provides a feasible method for future large-scale college student mental health screening and monitoring, providing policymakers with valuable insights for targeted interventions.

## Data availability statement

The raw data supporting the conclusions of this article will be made available by the authors, without undue reservation.

## Ethics statement

The studies involving human participants were reviewed and approved by Xuzhou Medical University. The patients/participants provided their written informed consent to participate in this study. Written informed consent was obtained from the individual(s) for the publication of any potentially identifiable images or data included in this article.

## Author contributions

HX and XW performed the majority of the study and conducted experiments. XL reviewed the data, revised the manuscript, and designed the overview framework. All authors approved the final version of the manuscript.
